# Engineered superparamagnetic iron oxide nanoparticles (SPIONs) for dual-modality imaging of intracranial glioblastoma via EGFRvIII targeting

**DOI:** 10.3762/bjnano.10.181

**Published:** 2019-09-11

**Authors:** Xianping Liu, Chengjuan Du, Haichun Li, Ting Jiang, Zimiao Luo, Zhiqing Pang, Daoying Geng, Jun Zhang

**Affiliations:** 1 Department of Radiology, Huashan Hospital, Fudan University, 12 Wulumuqi Middle Road, Shanghai 200040, China; 2 School of Pharmacy, Fudan University, Key Laboratory of Smart Drug Delivery, Ministry of Education, 826 Zhangheng Road, Shanghai 201203, China

**Keywords:** epidermal growth factor receptor variant III (EGFRvIII), glioblastoma, magnetic resonance imaging (MRI), molecular imaging, superparamagnetic iron oxide nanoparticles (SPIONs), nanomedicine, tumor resection

## Abstract

In this work, a peptide-modified, biodegradable, nontoxic, brain-tumor-targeting nanoprobe based on superparamagnetic iron oxide nanoparticles (SPIONs) (which have been commonly used as *T*_2_-weighted magnetic resonance (MR) contrast agents) was successfully synthesized and applied for accurate molecular MR imaging and sensitive optical imaging. PEPHC1, a short peptide which can specifically bind to epidermal growth factor receptor variant III (EGFRvIII) that is overexpressed in glioblastoma, was conjugated with SPIONs to construct the nanoprobe. Both in vitro and in vivo MR and optical imaging demonstrated that the as-constructed nanoprobe was effective and sensitive for tumor targeting with desirable biosafety. Given its desirable properties such as a 100 nm diameter (capable of penetration of the blood–brain barrier) and bimodal imaging capability, this novel and versatile multimodal nanoprobe could bring a new perspective for elucidating intracranial glioblastoma preoperative diagnosis and the accuracy of tumor resection.

## Introduction

Tumor resection is one of the most promising clinical treatments of glioblastoma, which is commonly associated with high mortality and inevitable tumor recurrence. To achieve a complete excision of tumors, the development of methods for accurate and efficient preoperative diagnosis and localization of glioblastoma is highly needed. Molecular imaging of tumor biomarkers is a powerful and important clinical diagnostic tool for noninvasive glioblastoma detection and characterization. Precise tumor resection is critical for affected patients and allows for better prognosis due to the infiltrative and heterogeneous characterization of glioma [1]. Glioma originates from glial cells and is a malignant tumor of the brain that exhibits hypervascularity, especially the grade IV, glioblastoma multiform (GBM) [2–3]. Complete excision of the tumor relies on the accurate preoperative diagnosis and precise intraoperative localization of lesions during surgery. Magnetic resonance (MR) imaging is an essential clinical imaging method for accurate diagnosis of central nervous system (CNS) diseases, such as glioblastoma [4]. On one hand, given the lack of sensitivity in conventional MR imaging, the development of molecular MR imaging of tumor biomarkers is highly urgent for noninvasive, visual presentation of cancer aggressiveness and guidance of glioblastoma excision [5]. In addition, gadolinium (Gd)-based agents (often Gd-diethylenetriaminepentaacetic acid (DTPA)) and superparamagnetic iron oxide nanoparticles (SPIONs) are the paramagnetic materials generally used as contrast agents to impact the relaxation time *T*_1_ or *T*_2_, thus generating bright or dark images via MR imaging. Gd-DTPA, as a commonly used positive contrast agent, despite its extensive application in *T*_1_-weighted MR imaging, has the disadvantages of unpredictable renal toxicity [6] and limited blood halftime [7]. Meanwhile, SPIONs, often used as negative contrast agents in MR imaging [8–10], have been extensively studied with regard to their long blood half-life [11], easy surface modification and excellent biocompatibility [12]. In the past years, several multifunctional vehicles for glioblastoma imaging have been developed, such as gold nanoparticles [13–14], which were integrated for diagnosis and treatment. Recently, the development of nanotechnology has made SPIONs promising candidates as molecular MR imaging probes as well [15–18].

The epidermal growth factor receptor variant III (EGFRvIII), the most common mutation type of the epidermal growth factor receptor (EGFR), is highly overexpressed in malignant tumors (about 25% to 64% in glioblastoma), which may contribute to the aggressive and refractory course of GBM [19–20]. Clinical evidence shows that EGFRvIII overexpression is associated with poor prognosis in patients suffering from glioblastoma [21–25]. Thus, EGFRvIII is a promising marker for sensitive glioblastoma detection and localization in molecular MR imaging. PEPHC1 (the sequence: HFLIIGFMRRAACGA) is a small peptide that has been identified for specific binding with EGFRvIII [26]. Thus PEPHC1 grafting to PEGylated SPIONs could help to construct a targeted nanoprobe for sensitive characterization and detection of glioblastoma.

Compared with single mode imaging, multimodal imaging can facilitate early cancer detection, providing a more comprehensive and multidimensional description of tumor biological behavior [27–29]. Optical imaging enables the visualization of pathophysiological processes with high sensitivity but with relatively low spatial resolution and shallow penetration into the tissue [30–31]. Cyanine7.5 NHS ester (Cy7.5), a near infrared fluorescence dye, has attracted extensive attention from researchers in various fields, including optical imaging [32–34].

There are many targeted probes for the diagnosis and treatment of GBM that have been constructed with high expression of EGFRvIII in the literature. Hadjipanayis and co-workers showed the specific EGFRvIII targeting and MRI contrast enhancement by means of EGFRvIII antibody-functionalized iron oxide nanoparticles after convection-enhanced delivery (CED) [35]. Lee and co-workers also showed that combining MRI with fluorescent imaging by using fluorescent silica-coated iron oxide nanoparticles has potential application in GBM treatment for improved intraoperative staging and enhanced radical surgery [36]. In addition, Mao and co-workers have reported the multitargeted drug delivery system by a d-peptide ligand (d-AE) based EGFRvIII targeting strategy, which provides a promising path for glioma therapy [37].

Through the conjugation of Cy7.5 to PEPHC1-modified PEGylated SPIONs, a multimodal imaging nanoprobe, which has both high sensitivity and high spatial resolution for early and precise detection and localization of glioblastoma, is constructed in this work. The multimodal imaging of EGFRvIII overexpression in glioblastoma was performed using Cy7.5-labeled SPIONs with EGFRvIII-targeting peptide PEPHC1. In vitro experiments and in vivo assessments on tumor-bearing nude mice were performed to demonstrate the multimodal imaging ability and biocompatibility of the as-constructed nanoprobe. To the best of our knowledge, these multifunctional nanoagents are reported herein for the first time to be used for the sensitive molecular MR and optical imaging of glioblastoma, resulting in significantly reduced doses of contrast agents needed in molecular MR imaging.

## Materials and Methods

### Materials

SPIONs (15 nm) were kindly donated by Prof. Shun Shen, Tongji University. PEPHC1 (C6-HFLIIGFMRRAACGA) peptide and 5-FAM-labeled PEPHC1 (5-FAM-C6-HFLIIGFMRRAACGA) peptide were synthesized by the Chinese Peptide Company (Hangzhou, China). EGFRvIII (D6T2Q) XPRabbit monoclonal antibody was purchased from Cell Signaling Technology (USA). Alexa Fluor 647-conjugated donkey-anti-rabbit secondary antibody was purchased from Abcam (USA). mPEG-DSPE (*M*_W_ = 2000) and DSPE-PEG-NH_2_ (*M*_W_ = 3400) was purchased from Laysan Bio (USA). DSPE-PEG-NHS (*M*_W_ = 2000) was obtained from Nanocs (USA). DAPI (2-(4-amidinophenyl)-1*H*-indole-6-carboxamidine) was obtained from Beyotime (Nantong, China). Coumarin-6 was bought from Sigma (USA) and Cy7.5 NHS ester was purchased from Nanocs (USA). Fetal bovine serum (FBS), phosphate buffered saline (PBS), trypsin-EDTA (0.25%), high glucose Dulbecco’s modified Eagle’s medium (DMEM) and penicillin-streptomycin were purchased from Gibco (CA, USA). The MTT cell proliferation and cytotoxicity assay kit was obtained from Sigma (USA). Puromycin was purchased from Aladdin (Shanghai, China). All other reagents were of analytical grade and used without further purification.

### Cells and animals

The human glioblastoma cell line, U87MG, was purchased from The Institute of Biochemistry and Cell Biology, Shanghai Institutes for Biological Sciences, Chinese Academy of Sciences (Shanghai, China). The U87MG-EGFRvIII cell line was purchased from the GeneChem (Shanghai, China). U87MG and U87MG-EGFRvIII cells were cultured in DMEM supplemented with 10% FBS and 1% penicillin/streptomycin at 37 °C in a humidified ecosystem containing 5% CO_2_.

Male Balb/c nude mice of 20 g were bought from SLAC Lab Animal Ltd. (Shanghai, China) and raised in SPF level environments. The intracranial in situ glioblastoma model of mice was constructed in step with a slightly modified, previous report [38]. Briefly, 5 × 10^5^ U87MG-EGFRvIII cells suspended in 5 μL of PBS was inoculated into the right striatum of nude mice with a stereotactic fixation device (Stoteling, USA). The location of injection was as follows: 2 mm right lateral to the bregma and 4 mm depth from the dura. All animal involved in this study were maintained according to the experimental protocol approved by the Ethics Committee of Fudan University.

### Synthesis of PEG-SPIONs conjugated with (PNPs) and without (NPs) PEPHC1

DSPE-PEG-Cy7.5 was synthesized through the reaction of the terminal NHS group of Cy7.5 with the NH_2_ group of the bifunctional PEG derivative. Briefly, Cy7.5/DSPE-PEG-NH_2_ 2:1 was dissolved in 10 mL of anhydrous dichloromethane and then reacted for 30 min. Then, the combination was evaporated with a ZX-98 rotary evaporator (Shanghai Institute of Organic Chemistry, China) for 1 h at room temperature. Cy7.5- and coumarin-6-decorated PEG-SPION (labeled as NPs in this work) were prepared in line with an adapted procedure previously described [27]. In brief, 2 mg of SPIONs, 10 mg of mPEG-DSPE, 1 mg of DSPE-PEG-NHS, 1 μg coumarin-6 and 0.5 mg DSPE-PEG-Cy7.5 were combined in 10 mL of dichloromethane and sonicated (53 W) for 10 min at room temperature. After evaporation of the dichloromethane, the NPs were placed in 4 mL of PBS and sonicated (53 W) for 15 min at room temperature, followed by ultrafiltration (Millipore, 100 kDa cut-off) of the prepared samples in order to separate the free mPEG-DSPE, DSPE-PEG-NHS, coumarin-6 and DSPE-PEG-Cy7.5.

For the preparation of PEPHC1-decorated PEG-SPIONs (labeled as PNPs in this work), PEPHC1 (50 µL, 20 mg/mL) was added to the NP suspension (4 mL, 0.25 mg of Fe /mL) to react for 2 h at room temperature and filtered as described above. Finally, the purified PNP nanoprobes were stored at 4 °C until use.

To analyze the concentration of peptide in the supernatant, high-performance liquid chromatography (HPLC) (Agilent 1200, USA) was used, where the mixture of solvent A and solvent B (59/41, v/v) was used as the mobile phase. Solvent A was 0.1% trifluoroacetic acid dissolved in deionized water and solvent B was 0.09% trifluoroacetic acid with 80% acetonitrile solution. The peptide conjugation efficiency was calculated according to the following formula:


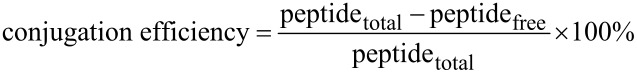


### Characterization of PEG-SPIONs

The morphological shape of the PNPs was analyzed by transmission electron microscopy (TEM) (H-600, Hitachi, Japan), followed by negative staining with 2% phosphotungstic acid. The mean hydrodynamic diameter and zeta potential of the nanoprobes were measured with a Malvern Zetasizer Nano ZS (Malvern, UK) instrument. The quantitative measurement of the Fe content in PNPs was conducted by inductively coupled plasma spectrometry (ICP-MS, Thermo Scientific iCAP 7400 series) [39]. *T*_2_-weighted MR imaging of the PNPs with various Fe concentrations was performed under a 3.0T clinical MRI scanner (DiscoveryMR750, GE Medical System, LLC, USA) at room temperature [27]. Fluorescent images of the PNPs with various Fe concentrations were acquired on an IVIS spectrum imaging application (PerkinElmer, USA) instrument and studied in the Living Imaging Software (PerkinElmer, USA).

### Validation of EGFRvIII expression on U87MG-EGFRvIII cells and PEPHC1 peptide binding with U87MG-EGFRvIII cells

U87MG and U87MG-EGFRvIII cells were seeded into 24-well plates and maintained for 24 h. When the cells reached 80% confluence, they were rinsed with PBS, then stained with EGF Receptor vIII rabbit monoclonal antibody (1 µg/mL) and Alexa Fluor 647-conjugated donkey-anti-rabbit secondary antibody (15 µg/mL). After completing the above steps, a qualitative examination of EGFRvIII expression on U87MG and U87MG-EGFRvIII cells was performed where the cells were stained with DAPI (1 µg/mL) for 5 min followed by imaging under the confocal microscope (ZEISS, 710, LSM, Germany). Then a quantitative analysis of the EGFRvIII expression on U87MG and U87MG-EGFRvIII cells was carried out on cells digested and gathered through centrifugation at 1000 rpm for 4 min. Finally, 0.5 mL of PBS (0.01 M, pH 7.4) was used to suspend the cells and the fluorescence intensity of the cells was ascertained by flow cytometry (BD, USA).

U87MG and U87MG-EGFRvIII cells were seeded into 24-well plates and cultured for 24 h. When the cells reached 80% confluence, they were incubated with 5-FAM-labled PEPHC1 (1 mg/mL) for 1 h. For fluorescence imaging, the culture solution was discarded, and the cells were incubated with 4% paraformaldehyde solution for 15 minutes at room temperature. Then the cells were stained with DAPI (1 µg/mL) for 5 min at room temperature followed by imaging with a confocal microscope (same as mentioned above). For the quantitative analysis of PEPHC1 peptide binding with U87MG and U87MG-EGFRvIII cells, the cells were digested, harvested and centrifugation was performed at 1000 rpm for 4 min. Finally, 0.5 mL of PBS (0.01 M, pH 7.4) was used to suspend the cells and the fluorescence intensity of the cells was ascertained by flow cytometry (BD, USA).

### Cellular uptake of PEG-SPIONs

The cellular uptake of NPs and PNPs were investigated on U87MG and U87MG-EGFRvIII cells. Briefly, U87MG and U87MG-EGFRvIII cells were seeded on 24-well plates (Corning Coaster, Japan) with a density of 1 × 10^4^ per well and cultured for 24 h. Subsequently, coumarin-6-labeled NPs and PNPs with the same Fe concentration (50 µg/mL) were added and incubated for 2 h at 37 °C. For the qualitative analysis, the cells were washed with PBS (0.01 M, pH 7.4) three times, then DAPI-stained and observed under a fluorescence microscope (Leica, DMI4000B, Germany). For the quantitative examination of the cellular uptake of the nanoprobes, U87MG and U87MG-EGFRvIII cells were harvested after trypsin digestion and suspended in 0.5 mL of PBS (0.01 M, pH 7.4) and analyzed with flow cytometry (BD, USA) at 488 nm.

### In vivo *T*_2_-weighted magnetic resonance imaging (MRI) of intracranial glioblastoma with PEG-SPIONs

Intracranial glioblastoma models were established as previously described [40–41]. In brief, 5 × 10^5^ U87MG-EGFRvIII cells mixed with 5 μL of PBS were injected into the right striatum (2 mm right lateral to the bregma and 4 mm depth from the dura) of male Balb/c nude mice with a stereotactic fixation device (Stoteling, USA). *T*_2_-weighted MRI was initially conducted to examine the location and size of the tumor two weeks after tumor-cell inocculation. Tumor-bearing mice were then randomly divided into three groups (NP, PNP and control group, *n* = 4) and received an injection of NPs, PNPs (20 mg of Fe/kg) or an equal volume of saline via the tail vein, respectively. Subsequently, *T*_2_-weighted MR images were obtained at various time points (8 and 24 h) after administration by a clinical 3.0T MR scanner (Discovery MR750, GE, USA) with a mouse special coil. The parameters for *T*_2_-weighted MR imaging are as follows: base resolution 256 × 128, field of view (FOV) 8 × 8 mm, slice thickness 1.2 mm, multiple echo times (TE) 8 ms, 16 ms, 24 ms, 32 ms, 40 ms, 48 ms, 56 ms, 64 ms, repetition time (TR) 1500 ms. The quantitative assay of the signal intensity was measured at the center of the tumor area with an operator-defined region of interest (ROI).

### In vivo fluorescence imaging

Two weeks after U87MG-EGFRvIII cell inoculation, the glioblastoma-bearing mice were randomly divided into 2 groups (NP and PNP group, *n* = 4). The accumulation of NPs and PNPs in the tumor tissue of glioblastoma-bearing mice was assessed as formerly described [42]. The mice were injected with Cy7.5-labeled NPs or PNPs at a dose of 20 mg of Fe/kg and fluorescence imaging was conducted at various time points (2 h, 4 h, 8 h, and 24 h) after intravenous injection using an IVIS spectrum imaging system (PerkinElmer, USA). The excitation wavelength was 788 nm and the emission wavelength of 808 nm of Cy7.5 was selected. 24 hours after nanoprobe injection, the tumor-bearing mice were sacrificed according to the previously described methods using heart perfusion with saline and the major organs (brain, heart, liver, spleen, lung and kidney) were sampled for ex vivo fluorescence imaging by an IVIS spectrum imaging system [27].

### Distribution of NPs and PNPs in tumor slices

To assess the tumor penetration effect and targeting characteristics of NPs and PNPs in vivo, the distribution of NPs and PNPs in glioblastoma slices was investigated. Two weeks after U87MG-EGFRvIII cell inoculation, the glioblastoma-bearing mice were randomly divided into two groups (NP and PNP group, *n* = 4) and were injected with 200 µL of Cy7.5-labeled NPs or PNPs at a dose of 20 mg of Fe/kg via the tail vein. 24 hours after injection, the mice were sacrificed and the brains were collected and dehydrated in 15% and 30% sucrose, embedded and cut into 10 µm sections. After staining with DAPI, fluorescence images of the brain slices were obtained with a laser confocal microscope (ZEISS, 710, LSM, Germany).

For electron microscopy samples, the tumor-bearing mice were sacrificed by heart perfusion with saline and 4% paraformaldehyde 24 hours after injection. Subsequently, the brains of the tumor-bearing mice were separated and the tumor tissue were removed and immersed in 2.5% glutaraldehyde for 2 h at 4 °C, followed by washing with PBS and the remaining steps as previously reported [27].

### Primary safety evaluation of PNPs

The cytotoxicity of PNPs against U87MG and U87MG-EGFRvIII cells was measured by a typical MTT assay. Briefly, U87MG and U87MG-EGFRvIII cells in the logarithmic growth phase were seeded in 96-well plates with a density of 1 × 10^4^ cells/well. After 24 h of incubation, the medium was discarded and 200 µL of fresh medium including NPs or PNPs with various Fe concentrations of 0, 25, 50, 100 and 200 µg/mL was added. After 24 h of incubation, the medium was discarded and 200 µL of fresh medium containing 20 µL of MTT solution was added to each well. After incubation for another 4 h, the medium was discarded and 150 µL of dimethyl sulfoxide (DMSO) solution was added to each well. Finally, the absorbance of each well was measured with a microplate reader (Bio-TEK, USA) at a wavelength of 490 nm.

To evaluate the in vivo systematic toxicity of PNPs, the histological sections of major organs (e.g., heart, liver, spleen, lung and kidney) were collected at 48 h after PNP injection at a Fe concentration of 20 mg/kg every 2 days for 4 weeks and subjected to H&E staining and microscopic examination (Leica, Germany).

### Statistical analysis

Differences in the assessment between experimental groups was evaluated using an unpaired student’s *t*-test. A value of *p* < 0.05 was considered statistically significant.

## Results and Discussion

### Preparation and characterization of PNPs

**Figure 1:**
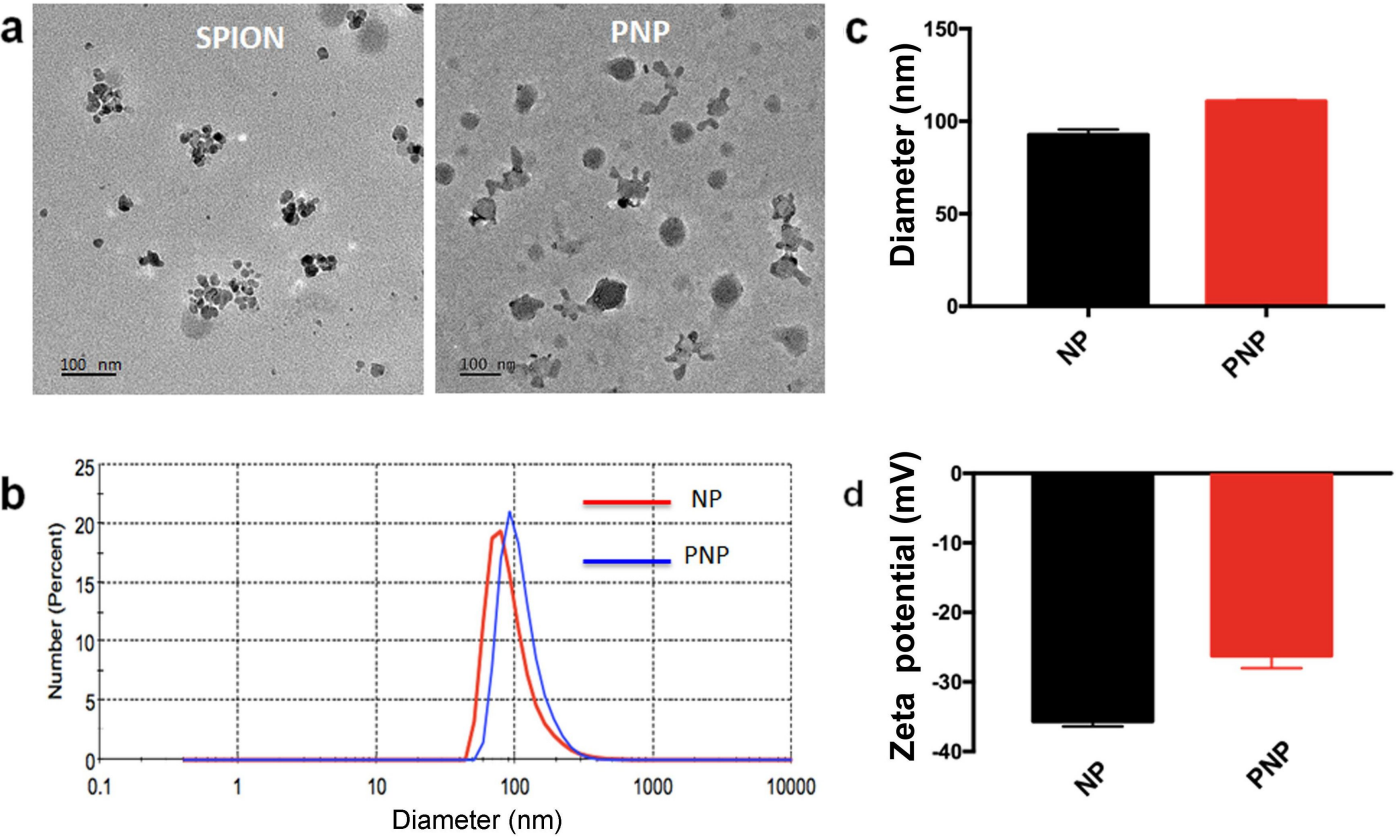
Characterization of PEG-SPIONs. (a) TEM images of SPIONs (left) and PEPHC1-conjugated NPs (right). (b) Dynamic light scattering (DLS) profile of NPs (before PEPHC1 conjugation) and PNPs (after PEPHC1 conjugation). (c) Hydrodynamic diameter of NPs and PNPs measured by DLS in water. (d) Zeta potential of NPs and PNPs.

A molecular-specific nanoprobe typically involves two major components: a signal component and a targeting moiety. In this work, we successfully constructed a brain-tumor-targeting nanoprobe which could be specifically accumulated in EGFRvIII-positive glioblastoma. The DSPE-PEG-Cy7.5 system was chosen as Cy7.5 is a widespread near-infrared dye with a long emission wavelength and has been applied extensively as a live imaging agent in the biomedical field. The as-synthesized DSPE-PEG-Cy7.5 material was then subsequently coated onto the hydrophobic SPIONs to make them hydrophilic and more stable for further fluorescent imaging experiments. Finally, PEPHC1, a small peptide that can specifically bind with the marker EGFRvIII that is overexpressed in glioblastoma, was grafted with the NHS group of DSPE-PEG-NHS through the amino group. The resultant PNP nanoprobe was characterized by TEM and DLS. As observed by TEM, the SPIONs suspended in hexane (Figure 1a (left)) had a diameter of approximately 15 nm and the PNPs (Figure 1a (right)) had a diameter of approximately 60 nm. The mean hydrodynamic diameter of the PNPs as measured by DLS in water was approximately 110.8 ± 0.4 nm with a polydispersity index (PDI) of 0.194. The diameter of the NPs was around 92.6 ± 1.7 nm with a PDI of 0.238 (Figure 1b,c) as determined by DLS. The DLS measurements demonstrated that the hydrodynamic particle size of whole clusters was around 100 nm, which is much larger than the diameter as determined by TEM. This discrepancy might be due to nanoprobe cluster formation in water. Nanoparticles of diameter around 110 nm can successfully escape the phagocytosis of the reticuloendothelial system (RES) and accumulate in tumor tissue under the influence of enhanced permeability and retention (EPR) as previously discussed in the literature [43–44]. The zeta potential of the NPs and PNPs was found to be around −35.6 mV and −26.2 mV, respectively. The less negative value of the PNP zeta potential as compared to the NP zeta potential confirmed the successful conjugation of the positive-valued targeting peptide PEPHC1 (Figure 1d). High-performance liquid chromatography (HPLC) determination (results not shown) further supported the successful conjugation of PEPHC1 where the conjugation efficiency of the PEPHC1 peptide on PNPs was calculated to be as high as 45.3%. Under our experimental conditions, it could be calculated that there were 240 PEPHC1 peptides for each PNP.

**Figure 2:**
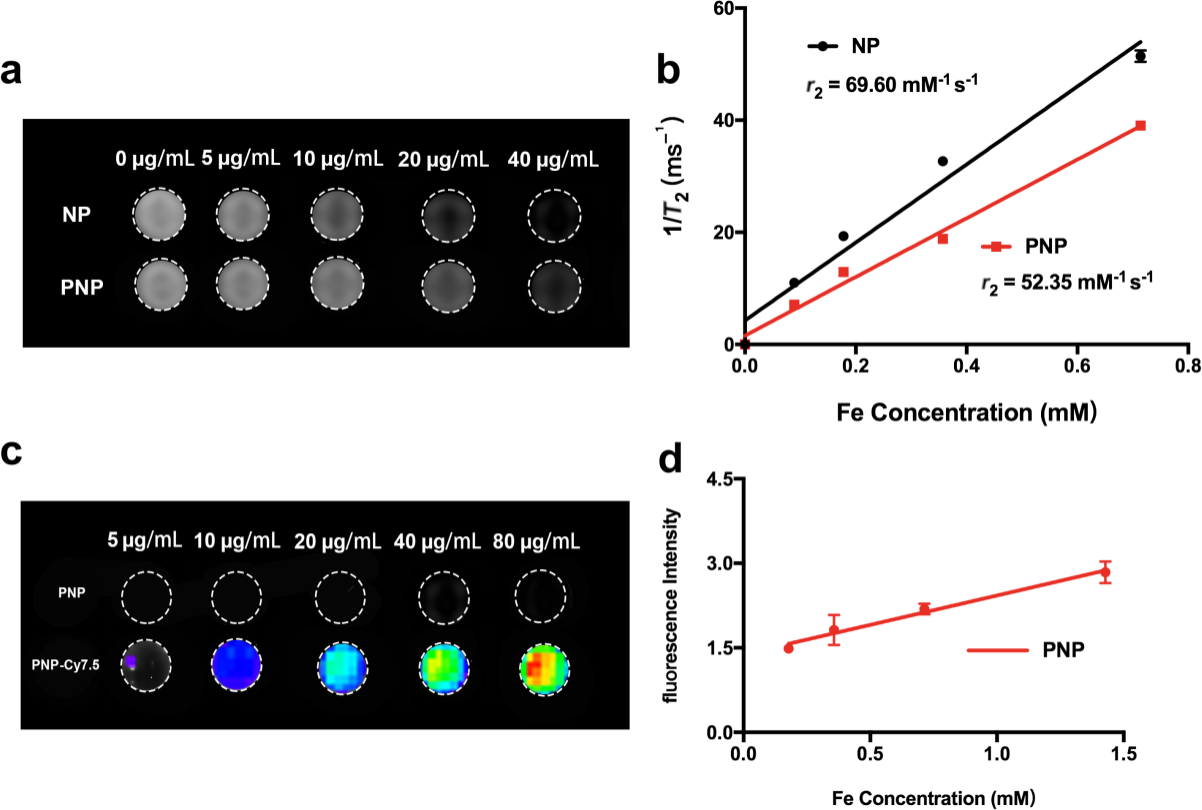
*T*_2_-weighted MR and fluorescence imaging of NPs and PNPs in vitro. (a) *T*_2_-weighted MR images of NPs and PNPs with Fe concentration given. (b) 1/*T*_2_ plotted against Fe concentration for NPs and PNPs. (c) Fluorescence images of PNPs acquired on an IVIS spectrum imaging system as a function of Fe concentration. (d) Fluorescence intensity of PNPs plotted as a function of Fe concentration.

The *T*_2_-weighted MR images of the PNP nanoprobes were evaluated in vitro with a 3.0 T clinical MRI scanner. As demonstrated in Figure 2a, the MR signal intensities of NPs and PNPs decreased with increasing Fe concentration (Figure 2a). After linear fitting, a good linear correlation between *T*_2_ relaxation (*r*_2_) and the Fe concentration was established. The *r*_2_ of the PNP nanoprobe was 52.35 mM^−1^ s^−1^, which was slightly lower than that of the NPs (69.60 mM^−1^ s^−1^). Both NPs and PNPs were appropriate for *T*_2_-weighted MR imaging because of their strong magnetization due to their superparamagnetic behavior (Figure 2b). In order to evaluate the fluorescent imaging ability of PNPs, fluorescent images of PNPs with various Fe concentrations were acquired. The results showed that a discernible PNP fluorescent signal was found at 5 μg/mL (Fe concentration) (Figure 2c), and the fluorescence intensity correlated linearly with Fe concentration (Figure 2d). Therefore, the as-synthesized PNP nanoprobes possessed outstanding properties for utilization as multifunctional vehicles for biomedical applications.

### EGFRvIII expression in U87MG-EGFRvIII cells

**Figure 3:**
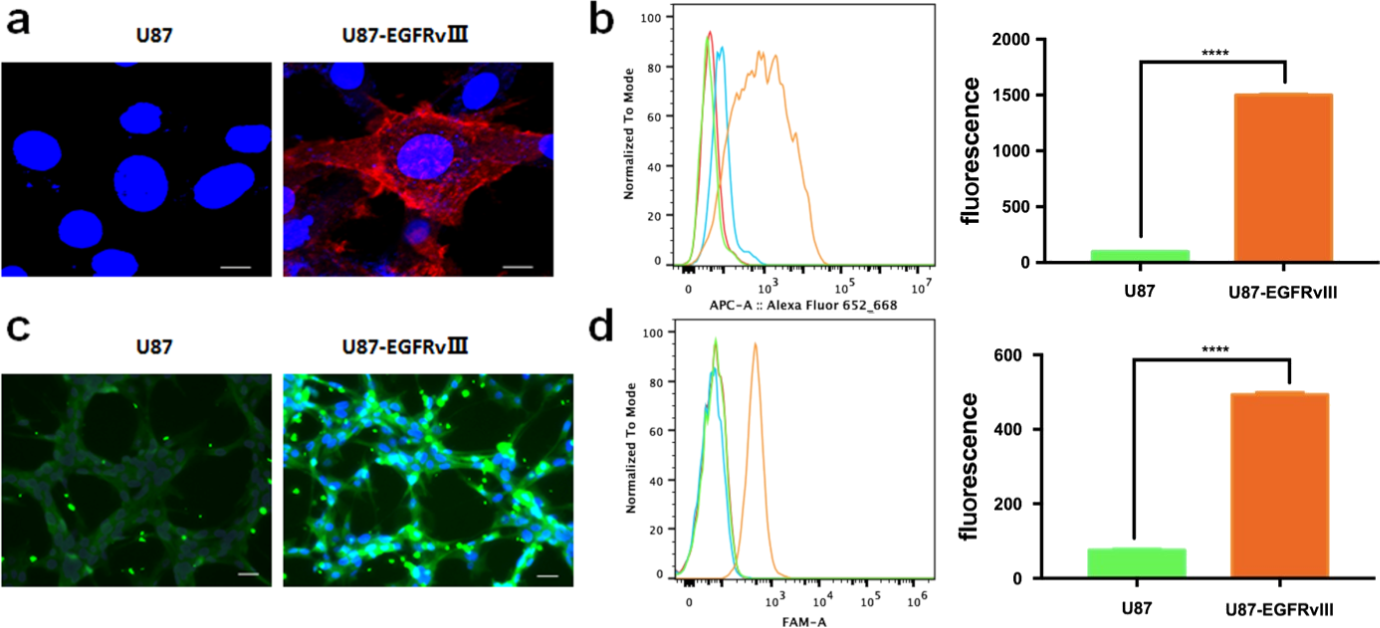
EGFRvIII expression and PEPHC1 binding with U87MG and U87MG-EGFRvIII cells. (a) Fluorescence images of U87MG and U87MG-EGFRvIII cells stained with EGFRvIII antibody. The nuclei were stained in blue and EGFRvIII was stained in red. (b) FACS assay (left) and quantitative analysis of EGFRvIII expression in U87MG and U87MG-EGFRvIII cells. (c) Fluorescence images of U87MG and U87MG-EGFRvIII cells after incubation with 5-FAM-labeled PEPHC1 peptide. The nuclei were stained in blue and PEPHC1 in green. (d) FACS assay (left) and quantitative analysis of PEPHC1 peptide binding to EGFRvIII in U87MG and U87MG-EGFRvIII cells. *** *p* < 0.0001 compared with U87.

Before assessing the uptake of the nanoprobes by different cells, EGFRvIII expression in U87MG-EGFRvIII cells was initially validated. As shown in Figure 3a, an obviously higher fluorescence intensity was observed in U87MG-EGFRvIII cells after immunostaining, while only a faint fluorescence intensity was detected in U87MG. In addition, a FACS assay further demonstrated that the expression level of EGFRvIII in U87MG-EGFRvIII cells was 15.5-fold that of U87MG (Figure 3b), which agreed well with the results observed by confocal microscopy (Figure 3a).

Subsequently, the targeting capability of the PEPHC1 peptide to U87MG-EGFRvIII cells was identified. After incubation with the peptide PEPHC1, U87MG-EGFRvIII cells demonstrated significantly higher fluorescence intensity than U87MG (Figure 3c). A further FACS assay revealed that the binding of PEPHC1 peptide with U87MG-EGFRvIII cells was 6.5-fold that of U87MG (Figure 3d), which agreed well with the results observed by confocal microscopy (Figure 3c). These results indicated that U87MG-EGFRvIII cells overexpressed EGFRvIII and PEPHC1 peptide had special targeting to U87MG-EGFRvIII cells.

### Cellular uptake of PNPs by U87MG-EGFRvIII cells

**Figure 4:**
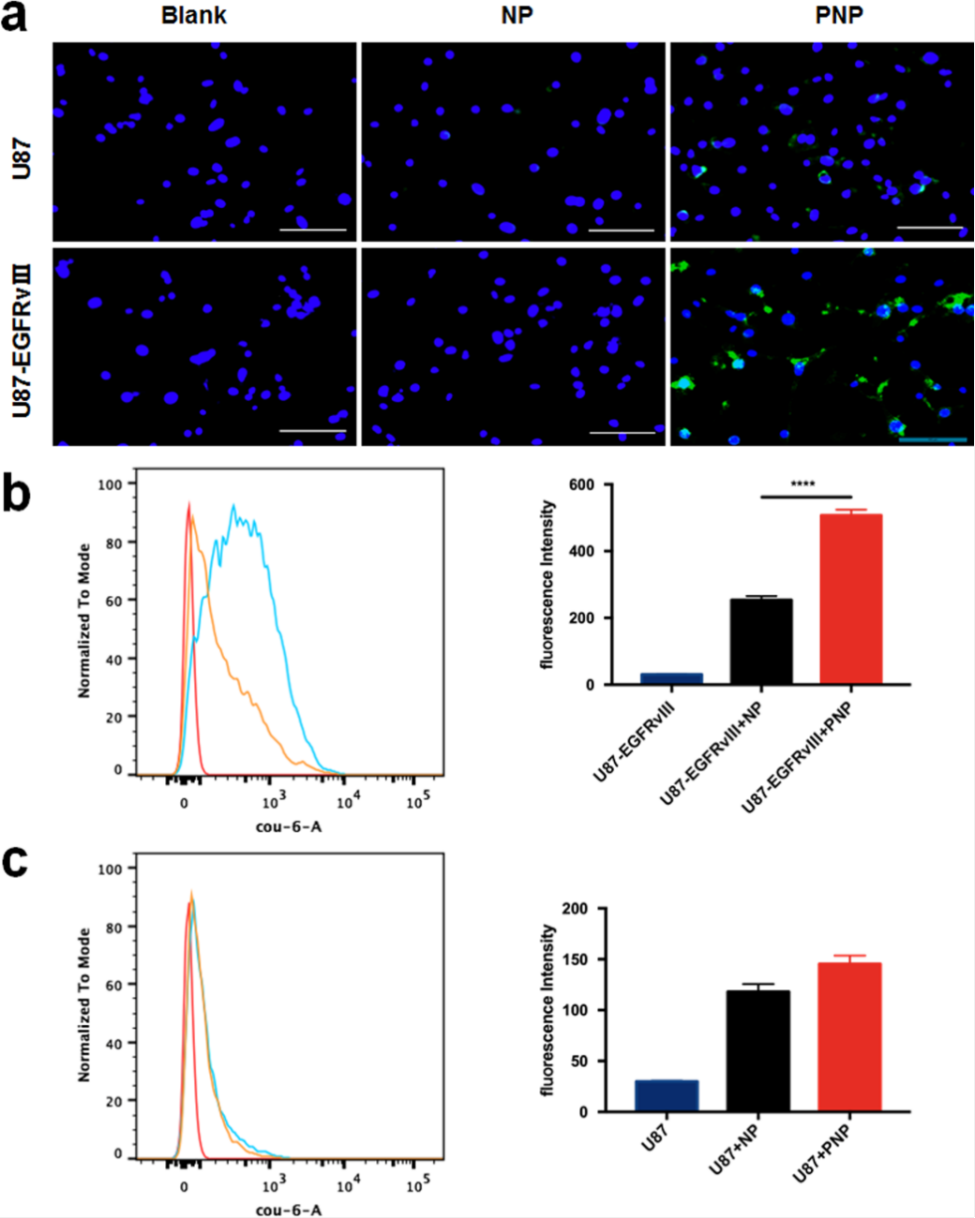
Cellular uptake of NPs and PNPs by U87MG and U87MG-EGFRvIII cells. (a) Fluorescence images of U87MG and U87MG-EGFRvIII cell uptake of cou-6-labeled NPs and PNPs after incubation for 0.5 h at 37 °C, respectively (scale bar = 100 µm). FACS assay (left) and quantitative analysis of cellular uptake of the nanoprobes by (b) U87MG-EGFRvIII and (c) U87MG cells. Untreated cells are shown as the blank. *** *p* < 0.0001 compared with U87MG-EGFRvIII+PNP.

To demonstrate the targeting ability of PNPs, in vitro cellular uptake of PNPs was investigated by U87MG-EGFRvIII cells. After incubation with NPs and PNPs for 2 h, the fluorescence intensity of U87MG-EGFRvIII cells in the PNP group was observed to be much stronger over the NP group (Figure 4a). The flow cytometry results further indicated that the accumulation of PNPs in U87MG-EGFRvIII cells was about 2.3-fold that of the NPs as shown in Figure 4b. In contrast, after incubation with NPs and PNPs for 2 h, there was no significant difference in the fluorescence intensity of U87MG cells of the PNP group compared with that of the NP group (Figure 4a), which was further verified by flow cytometry assay (Figure 4c). These results suggested that the PEPHC1 peptide conjugation could significantly enhance the uptake of PNPs by U87MG-EGFRvIII cells but not by U87MG cells.

### In vivo magnetic resonance and fluorescence imaging

**Figure 5:**
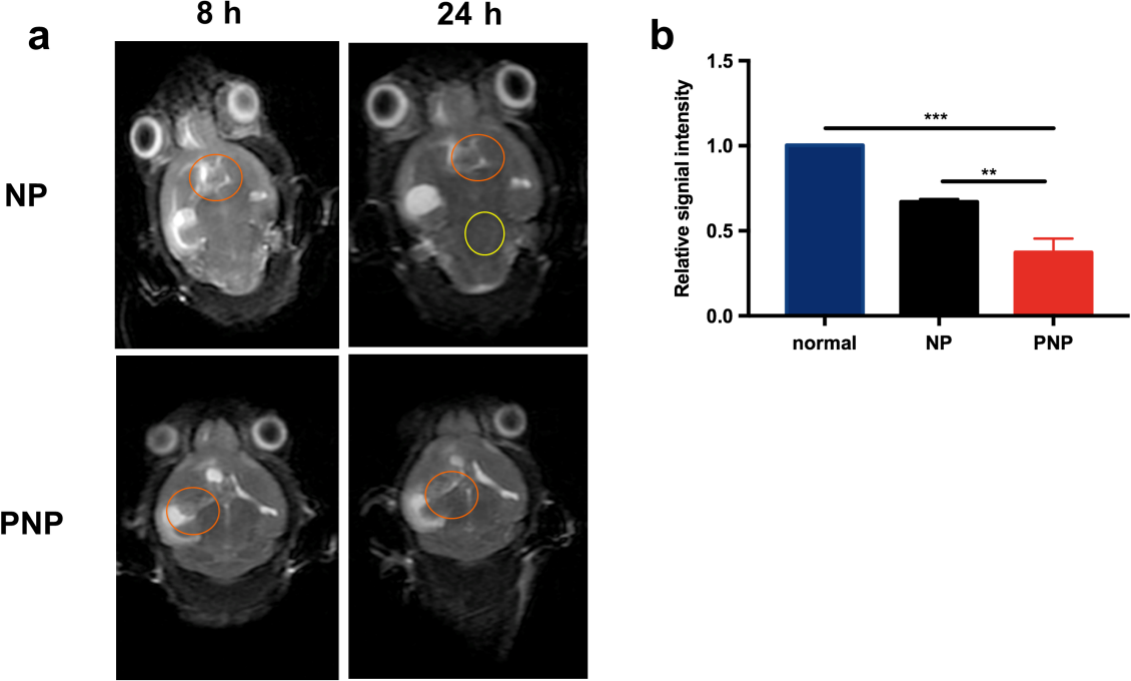
In vivo *T*_2_-weighted MR imaging of EGFRvIII-positive glioblastoma with PNPs. (a) *T*_2_-weighted MR imaging of tumor-bearing mice at different time points after NP or PNP injection. (b) The semi-quantitative measurements of signal intensity in the tumor ROI in *T*_2_-weighted MR images 24 h post injection. ** *p* < 0.01, *** *p* < 0.001 compared with PNPs.

The dual-modality imaging capability of PNPs for U87MG-EGFRvIII cells was tested in vivo. Two weeks after U87MG-EGFRvIII cell inoculation, the glioblastoma-bearing mice received NP or PNP injections, and *T*_2_-weighted MR imaging and was conducted at different time points. As shown in Figure 5a and 5b for the PNP group, there was a strong signal loss in the brain tumor region, demonstrating the targeted imaging capability of PNPs for U87MG-EGFRvIII tumors. On the contrary, the mice that were injected with the non-targeting NPs displayed only negligible signal changes in the brain tumor regions.

**Figure 6:**
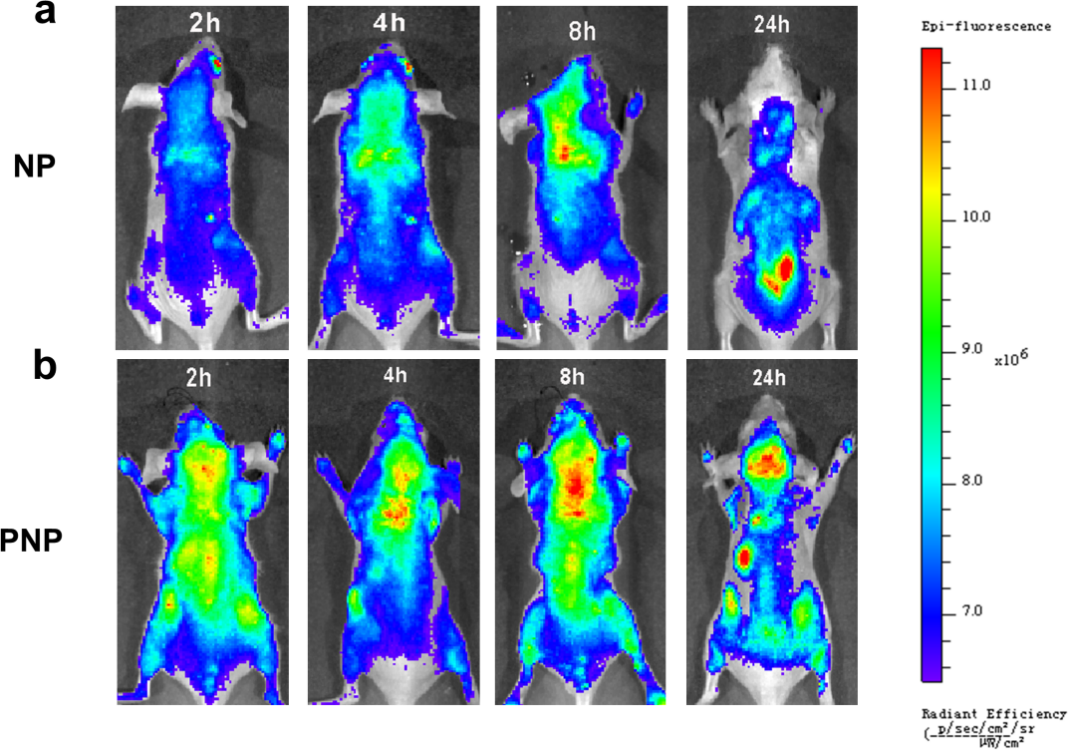
In vivo fluorescence imaging of EGFRvIII-positive glioblastoma at different time points after PNP or NP injection.

Moreover, fluorescence imaging revealed that the fluorescence intensity in the brain of the mice injected with PNPs was stronger than that of the mice injected with NPs, and the intensity peaked at 24 h (Figure 6a and 6b).

**Figure 7:**
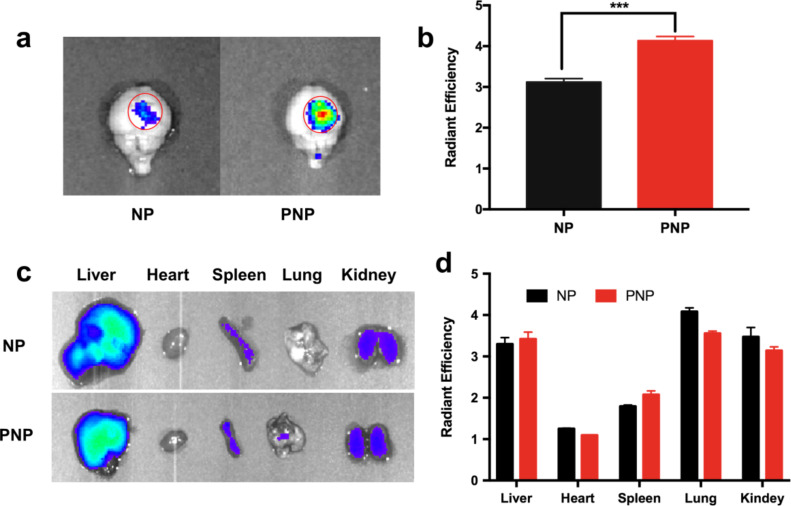
Ex vivo fluorescence imaging of brain and major organs. Fluorescence imaging (a) and intensity (b) of brains collected at 24 h after intravenous injection of NPs or PNPs, *** *p* < 0.001 compared with NPs. Fluorescence imaging (c) and intensity (d) of liver, heart, spleen, lung and kidney of tumor-bearing mice collected at 24 h after intravenous injection of NPs or PNPs.

To evaluate the distribution of these nanoprobes more precisely, the mouse brains were collected and ex vivo fluorescence imaging was conducted. Obviously, the fluorescence intensity in the brain tumor from the PNP group was significantly higher than the NP group 24 h post injection, indicating that the PEPHC1 peptide conjugation greatly enhanced PNP accumulation in U87MG-EGFRvIII tumors (Figure 7a and 7b). For the biodistribution study, similar to other drug delivery systems based on tumor-targeting nanoparticles [45–47], both PNP and NP nanoprobes were found distributed in other major organs, especially in the liver and kidney, indicating that the PEPHC1 peptide conjugation did not change the distribution of PEGylated SPIONs (Figure 7c and 7d). The in vivo environment was more complicated than under in vitro settings. Once introduced into the blood, nanoparticles are immediately adsorbed with proteins, resulting in the formation of protein corona [48–49]. To minimize the adverse effects of the presence of the protein corona in vivo, the surface coating using PEG can endow the NPs with so-called “stealth” properties to reduce the adsorption of high molecular weight proteins, allowing them to avoid the immune system. This system results in more stability in vitro and especially in vivo and allows the nanoprobe to achieve a long in vivo half-life [49].

### Distribution of PNPs in glioblastoma slices

**Figure 8:**
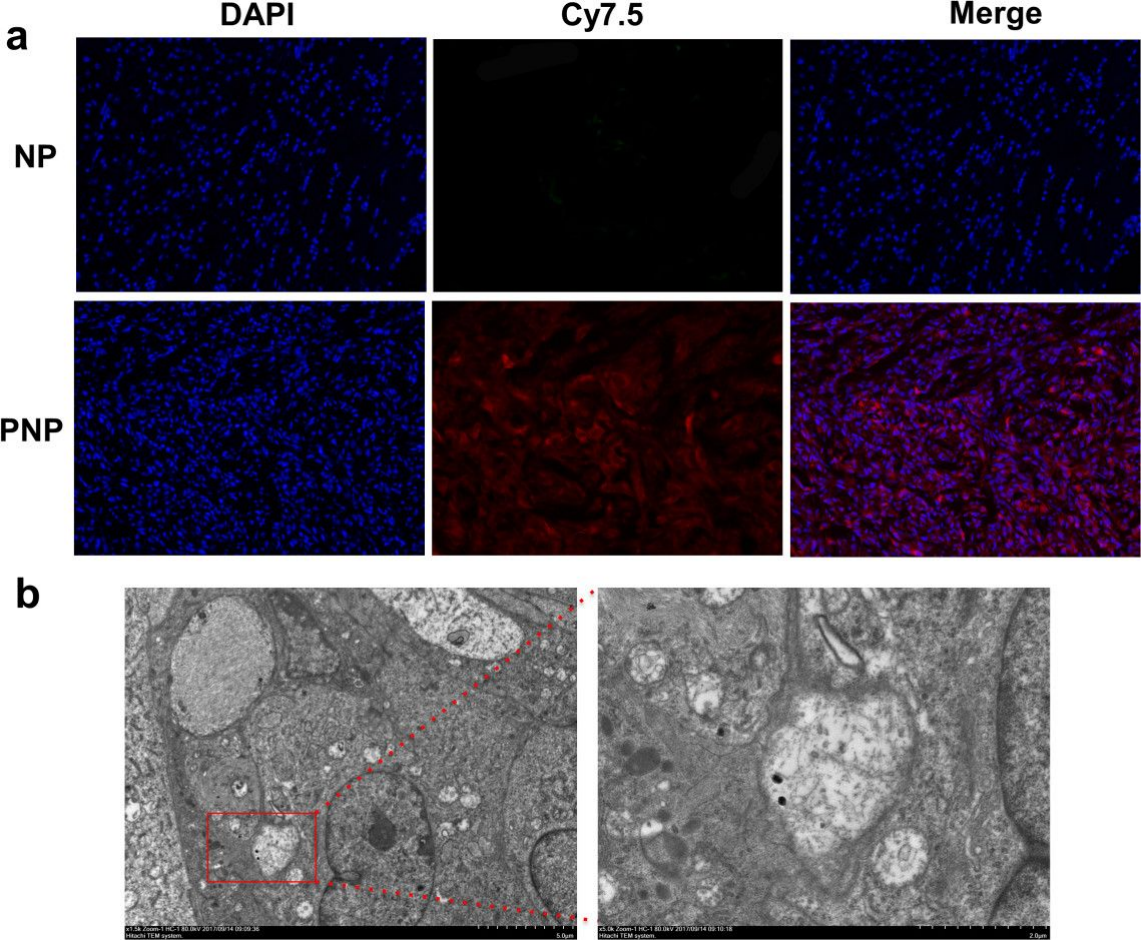
Distribution of PNPs and NPs in brain tumor slices. (a) Fluorescence images of U87MG-EGFRvIII tumor slices at 24 h after NP or PNP injection. (b) TEM images of PNPs in U87MG-EGFRvIII tumor slices at 24 h after PNP injection.

To further verify the specificity of the targeting capability of the PNPs, fluorescence images of tumor tissue of mice treated with PNPs and NPs 24 h after injection were taken. As demonstrated in Figure 8a, the PNPs penetrated deeply and accumulated more in the whole tumor with distinctly higher fluorescence intensity compared with NPs, demonstrating the contribution of the PEPHC1 peptide to the enhanced accumulation of PNPs in EGFRvIII-positive tumors. Further TEM imaging demonstrated that plenty of the PNP nanoprobes accumulated in U87-EGFRvIII cells, suggesting the increased endocytosis of PNPs in U87-EGFRvIII cells (Figure 8b).

### Primary safety evaluation of PNPs

**Figure 9:**
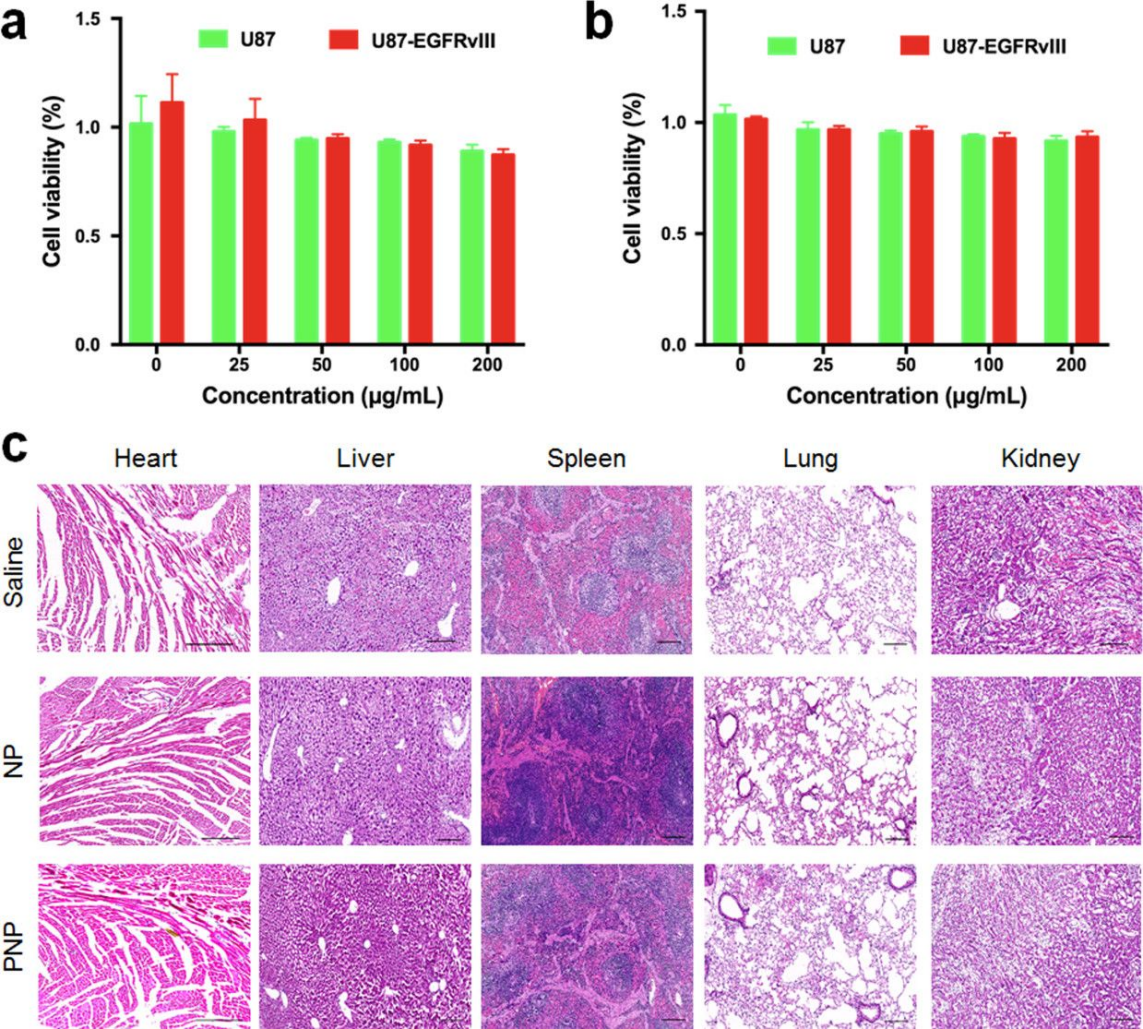
Cytotoxicity assays of (a) NPs and (b) PNPs against U87MG and U87MG-EGFRvIII cells. The cells were incubated with NPs or PNPs with different Fe concentrations for 24 h. (c) H&E staining of slices of heart, liver, spleen, lung and kidney from mice of different groups 48 h post PNP injection. No obvious necrosis could be observed in the three groups.

The cytotoxicity of nanoprobes is a primary consideration for clinical application. Therefore, the viability of U87MG and U87MG-EGFRvIII cells treated with NPs and PNPs with different concentrations was investigated using the MTT assays. Even at a high Fe concentration of 200 µg/mL, more than 95% of cell viability was found, indicating that both NPs (Figure 9a) and PNPs (Figure 9b) induced no obvious cytotoxicity, and thus possess favorable biocompatibility. Moreover, H&E staining of the major organs demonstrated no obvious lesions in both of the NP and PNP groups (Figure 9c), indicating that the PNPs had a favorable biosafety for molecular imaging.

A nanoprobe for the multimodal imaging of EGFRvIII overexpression in glioblastoma was constructed in the study. Compared with the EGFRvIII-antibody-functionalized iron oxide nanoparticles after convection-enhanced delivery (CED) to target EGFRvIII [35] and fluorescent-silica-coated iron oxide nanoparticles to target tumor-associated macrophages [36] and D-AE-peptide-modified micelles as a multitarget drug delivery system [37], the benefits of the nanoprobe in this study are described as follows. First, the nanoprobe has a diameter of around 100 nm and can directly pass through the blood–tumor barrier. Second, bimodal imaging combining magnetic resonance imaging and optical imaging provides a more accurate means for accurate diagnosis of glioblastoma. Third, the nanoprobe has good targeting to overexpressed EGFRvIII in glioblastoma and thus establishes a foundation for in vivo targeted therapy of glioblastoma.

## Conclusion

In summary, a novel dual-modal molecular imaging capability has been created through conjugating PEPHC1 to PEGylated SPIONs, aiming to improve the sensitivity of MR imaging and the spatial resolution of optical imaging. Both the in vitro and in vivo experiments demonstrated the excellent ability of the as-constructed nanoprobes to detect and characterize U87MG-EGFRvIII tumors with high sensitivity via MRI and optical imaging. Meanwhile, the systematic toxicity experiments indicated that PNPs are safe and can be used for further research. Therefore, based on these results, we can highly recommend that the as-constructed nanoprobes can be used as contrast agents to characterize intracranial EGFRvIII-positive glioblastoma via MRI and optical imaging and can improve the accuracy of surgical resection of the glioblastoma.
